# Association mining based approach to analyze COVID-19 response and case growth in the United States

**DOI:** 10.1038/s41598-021-96912-5

**Published:** 2021-09-20

**Authors:** Satya Katragadda, Raju Gottumukkala, Ravi Teja Bhupatiraju, Azmyin Md. Kamal, Vijay Raghavan, Henry Chu, Ramesh Kolluru, Ziad Ashkar

**Affiliations:** Informatics Research Institute, University of Louisiana at Lafayette, Lafayette, 70506 USA

**Keywords:** Public health, Epidemiology, Data mining

## Abstract

Containing the COVID-19 pandemic while balancing the economy has proven to be quite a challenge for the world. We still have limited understanding of which combination of policies have been most effective in flattening the curve; given the challenges of the dynamic and evolving nature of the pandemic, lack of quality data etc. This paper introduces a novel data mining-based approach to understand the effects of different non-pharmaceutical interventions in containing the COVID-19 infection rate. We used the association rule mining approach to perform descriptive data mining on publicly available data for 50 states in the United States to understand the similarity and differences among various policies and underlying conditions that led to transitions between different infection growth curve phases. We used a multi-peak logistic growth model to label the different phases of infection growth curve. The common trends in the data were analyzed with respect to lockdowns, face mask mandates, mobility, and infection growth. We observed that face mask mandates combined with mobility reduction through moderate stay-at-home orders were most effective in reducing the number of COVID-19 cases across various states.

## Introduction

COVID-19 outbreak has brought the world to a standstill. Until the COVID-19 vaccine became available recently, several non-pharmaceutical interventions were used to contain the outbreak, which included stay-at-home orders, social distancing at 6 feet, limited gatherings, hand washing, refraining from touching the face, and masking. Among these, stay-at-home orders potentially carried a high economic cost in lost revenue and financial support to the unemployed^1^. Given the complex dynamics of COVID-19, the high variability of intervention strategies, and the complexity of pandemic behavior, understanding the differential impact of combinations of various measures is non-trivial.

Several methods have been used to study the impact of various non-pharmaceutical interventions and policies on COVID-19 infection growth rate. The initial studies focused on agent-based simulations and statistical correlation analysis. Li et al. used a compartmental model to evaluate the effect of social distancing and cloth face coverings on the spread of infections^2^. Tatapudi et al. presented a study on Miami-Dade County to understand how social-mixing behavior, stay-at-home orders, and contact tracing affect both the case growth and economy^3^. Similar efforts include agent-based simulation developed by Silvia et al.^4^, and Ghaffarzadegan^5^. As more case growth data became available, researchers used data-driven methods to find associations between non-pharmaceutical interventions and case growth data. Correlation and regression-based analysis were performed by Badr et al.^6^ and Sarmadi et al.^7^ to understand the impact of mobility on infection spread. Bendavid et al. used regression-based analysis to study the impact business closures and stay-at-home orders on epidemic case growth across 10 countries, including the United States^8^. Regression-based methods were used to understand the association between the timing of mandated lockdown orders^9^, social, economic, and demographic determinants^10^, and the spread of COVID-19 across various counties in the United States. The dynamics of incidence and mortality rates were also found to vary across regions in the United States^11^. James and Menzies studied the second surge in COVID-19 cases to understand the evolutionary patterns using time-series analysis and hierarchical clustering. The second surge revealed common characteristics of states that were most and least successfully managed COVID-19^12^.

Several studies analyzed the impact of face mask usage on the number of COVID-19 cases. A recent study found that a universal mask mandate would help alleviate the worst effects of epidemic resurgence in many states across the United States^13^. Fischer et al. applied logistical regression-based models on mask-wearing and social distancing guidelines and found that states with mask adherence $\ge$ 75% had 140 fewer cases per capita than states with less than 75% for mask adherence^14^. Dasgupta et al. used Poisson regression models to examine associations between the implementation of community mitigation policies and identification of a county as a rapid riser and found that counties in states that closed for fewer days (0 to 59) and had no mask mandate at reopening had a higher probability of becoming a rapid riser county^15^. Another study on 198,077 participants across the United States used hazard ratio to find associations between community-level social distancing measures and individual face mask use with reduced risk of COVID-19 surge^16^. Krishnamachari et al. examined the impact of school closures, stay-at-home orders, and mask mandates based on the length of the mandate on cumulative incidence rates of COVID-19 in all states in the US using negative binomial regression^17^. Lyu and Wehby compared the case growth rate between states with and without mask mandates during the pandemic using a regression-based approach^18^. Guy et al. used weighted least-squares regression to measure the impact of various policies like mask mandates and on-premises dining across 38 states in the US with the change in the case and death rates before and after the implementation of the policies^19^. Most of these studies look at the adherence to masks or social distancing guidelines across various counties and states and its impact on the number of cases. Rather than analyzing the impact of one or two non-pharmaceutical interventions, it is important to analyze the association between the combination of multiple interventions and local infection dynamics. To accomplish this, this paper introduces an association mining approach to analyze similarities across various policies and infection rates in communities for various phases of the pandemic.

Association rule mining (ARM) is a common data mining technique used to discover similarities and dissimilarities among objects^20^. The approach was originally designed to obtain insights into consumer buying habits, such as understanding the groups of products customers would buy together^20^. The approach later garnered interest in many domains^21–24^. Recently in public health, ARM was used to analyze the relationship between environmental stressors and adverse human health impacts^25^.

We used an ARM approach to analyze how various non-pharmaceutical interventions contributed to infection growth. Rather than offer clear hypothesis-based objectives, the proposed technique provides insights into similarities and dissimilarities among various combination of policies and local conditions that led to an increase or decrease in infection rates. We use publicly available data collected from all 50 states to discover common patterns with respect to similarities between six different factors, namely stay-at-home-orders, face masks, population density, mobility, and infection rates on future infection rates across various states in the United States.

## Data and methods

Association mining allows us to perform a descriptive analysis of patterns between various factors known to influence infection growth rate and the actual infection growth rate. We specifically looked at population density, infection rate, face mask orders, stay-at-home orders, and mobility^6,26–29^.

### Association rule mining

Given a dataset containing a collection of records or transactions, each record comprises a set of categorical attributes. One of the attributes is the target attribute of interest. The association rule may be denoted by $A \Rightarrow B$, where *A* (the antecedent or LHS) and *B* (the consequent or RHS) are sets of various attribute-value pairs (also called itemsets), and are disjoint. The rule represents the hypothesis that when variables in *A* occur in the dataset, the variables in *B* also occur. Association mining generates a large number of rules from a given dataset. In a dataset with *m* attributes ($n-1$ antecedents and one consequent), each with *n* values, each can generate a maximum of $nm^{(n-1)}-1$ rules. However, not all rules are significant. The goal of this approach is to find rules that have high practical significance. To eliminate spurious rules, we use three measures: support, confidence, and lift. In addition, we also use the chi-squared test to measure the statistical significance of the association between the antecedent and the consequent.

Given two disjoint sets of attribute-value pairs *A* and *B*, and an association rule $A \Rightarrow B$; support of the rule refers to the number of records where the attribute-value pairs in either set *A* or *B* appear in the dataset relative to the total number of records (transactions or instances). This denotes the prevalence of the rule in the dataset. By definition, the support value is symmetric (i.e., support of both rules $A \Rightarrow B$ and $B \Rightarrow A$ are equal). Similarly, *support(A)* is the total number of records containing the itemset A to the total number of records in the dataset. The confidence of the rule $A \Rightarrow B$ measures the conditional probability of *B*, given *A*. Thus, the confidence measure for a given rule is asymmetric.

1 $$\begin{aligned}&support (A \Rightarrow B)= support(A \cup B)= {\frac{|set \; of \; records \; containing \; A \cap \; set \; of \; records \; containing \; B|}{total \; number \; of \; records} } \end{aligned}$$

2 $$\begin{aligned}&{support } (A)= {\frac{|set \; of \; records \; containing \; A|}{total \; number \; of \; records} } \end{aligned}$$

3 $$\begin{aligned}&{confidence (A \Rightarrow B)= \frac{support (A \cup B)}{support (A)} }\end{aligned}$$

*Lift* is the ratio between the observed support and the expected support between the independent variables *A* and *B*. A $lift > 1$ implies a greater degree of dependence whereas, a $lift < 1$ indicates negative dependence, and $lift = 1$ shows that *A* and *B* are independent. Lift is also a symmetric measure between the itemsets *A* and *B*.

4 $$\begin{aligned} lift (A \Rightarrow B)= \frac{support (A \cup B)}{support (A)\times support(B)} \end{aligned}$$

In addition to lift, the chi-squared test has also been used to measure the statistical significance level of the dependence between antecedent and consequent in association rules^30,31^. However, it should be noted that the chi-squared test, being a symmetrical measure, does not measure the dependence of the antecedent and consequent of a rule which is provided by confidence measure from Eq. (3). The chi-squared value of an association rule $A \Rightarrow B$ is defined by Alvarez^31^ as a factor of support, confidence, and lift measures and is provided below:

5 $$\begin{aligned} \chi ^2(A \Rightarrow B) = n (lift(A\Rightarrow B)-1)^2\frac{support(A\Rightarrow B)confidence(A\Rightarrow B)}{(confidence(A\Rightarrow B)-support(A\Rightarrow B))(lift(A\Rightarrow B)-confidence(A\Rightarrow B))} \end{aligned}$$

where *n* is the total number of transactions in the dataset. The association between the antecedent and the consequent is considered significant if the chi-squared value is greater than a threshold determined by the chi-squared distribution. For an association rule, the degrees of freedom for an association rule is one?.

In this paper, we model face-covering orders, social distancing orders, mobility, population density, case level, and the current incident phase as the contributing factors (i.e., the antecedent). The target variable (the consequent) is the future incident growth phase. One of the critical assumptions for ARM is that all the values of attributes are discrete. We discretized the numerical data used in the study (i.e., mobility, number of cases per capita) into five quantiles. We also discretized the continuous data of infection growth curve into five phases based on the logistic growth model.

### Data collection and preprocessing

Our study includes weekly aggregated data from all the 50 states within the United States between June 1st and November 15th, 2020. We start our data collection on June 1st because including earlier data may skew our analysis (only eight states had a mask mandate before June and most of the states were under lockdown^32^). We end our study period on November 15th before the start of the winter holiday season. Discretized attributes, values, and the frequency distribution of each attribute-value pair are presented in Table 1.

**Table 1:** Breakdown of all the attributes, their values, and the frequency of the attribute-value pairs.

Attribute	Data	Attribute-values	Frequency of the attribute-value pairs
Mask mandate	No mask	No mask	367
Mask mandate	County-wide	County-wide	179
Mask mandate	State-wide	State-wide	505
Mask mandate	Recommended	Recommended	9
Social distancing	Phase 0	Phase 0	229
Social distancing	Phase 1	Phase 1	152
Social distancing	Phase 2	Phase 2	156
Social distancing	Phase 3	Phase 3	277
Social distancing	Phase 4	Phase 4	226
Social distancing	Phase 5	Phase 5	20
Mobility levels	0–15%	Very low	212
Mobility levels	> 15–40%	Low	217
Mobility levels	> 40–50%	Medium	209
Mobility levels	> 50–60%	High	216
Mobility levels	> 60%	Very high	206
Population density	0–150	Low	609
Population density	> 150–300	Medium	273
Population density	> 300	High	168
Cases per capita	0–0.1%	Very low	220
Cases per capita	> 0.1–0.3%	Low	220
Cases per capita	> 0.3–1%	Medium	239
Cases per capita	> 1–2%	High	203
Cases per capita	> 2%	Very high	178
Current Incidence Phase	Early-growth	Early-growth	286
Current incidence phase	Fast-growth	Fast-growth	438
Current incidence phase	Decline	Decline	222
Current incidence phase	Steady-state	Steady-state	95
Current incidence phase	End state	End state	9
Future incidence phase	Early-growth	Early-growth	313
Future incidence phase	Fast-growth	Fast-growth	419
Future incidence phase	Decline	Decline	231
Future incidence phase	Steady-state	Steady-state	80
Future incidence phase	End state	End state	7

#### Mask usage

**Figure 1:**
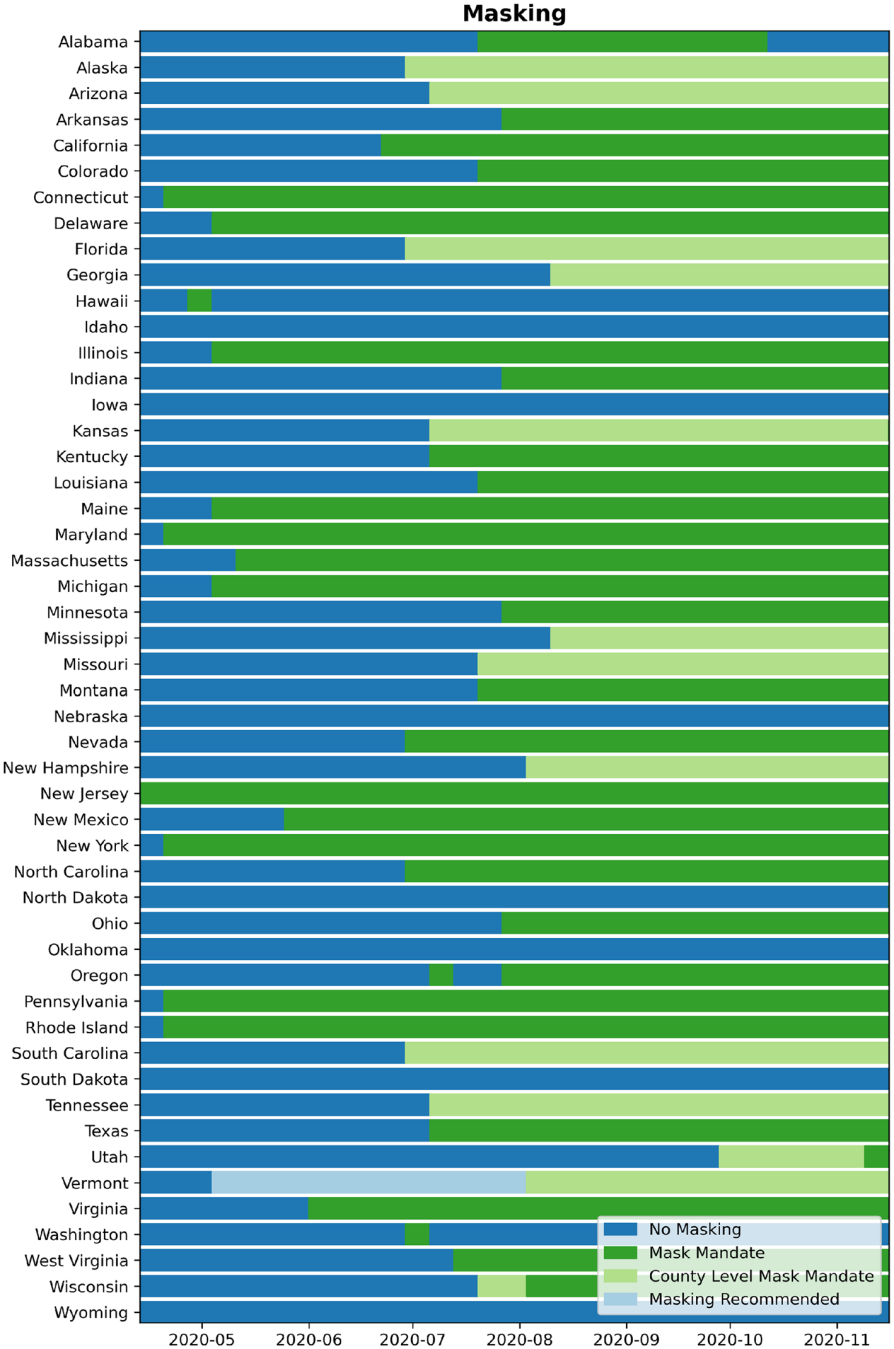
Timeline of various mask mandates issued across all the states in the United States.

We used the official face-covering orders issued by various governors or local authorities from AARP State-by-State Guide to Face Mask Requirements^33^ and Masks4All compilation^34^. We rounded the dates to the start of the workweek. The four categories of mask orders are No-Mask, county-wide, recommended (state-wide), and mandated (state-wide). The discretized dataset we produced and detailed definitions of each of these orders were provided on GitHub^35^. We illustrated the state mask mandate variation across all the states in Fig. 1.

#### State reopening

All states initiated a strict lockdown at the beginning of the pandemic in March 2020. The states modified these orders based on the perceived risk of cases, hospitalizations, and deaths while also trying to bring back the economy. States mostly adapted the guidelines provided by the White House COVID-19 task force reopening procedures^36,37^. The specific orders that were considered include Phase-0, Phase-1, Phase-2, Phase-3, Phase-4, and Phase-5. Detailed definitions of each of these orders were provided at this webpage^35^.

#### Mobility levels

The mobility information was from the Descartes Labs, a popular dataset used by several studies for analyzing the relationship between mobility and COVID-19 case growth^4,38,39^. The dataset uses anonymized mobile device locations to calculate a local mobility metric. The metric represents the median of the max-distance traveled by individuals at the state and county level normalized to the metric before the pandemic^40^.

#### Population density

The population density of each state represents the number of people per square mile of land area based on the 2020 population estimates^41^.

#### Cases per capita

We extracted the official COVID-19 weekly case data from June 1st to November 10th for the United States from the Johns Hopkins University Dashboard^42^. We calculated the per capita cases based on the estimated 2019 US Census population data.

#### Incidence phases

**Figure 2:**
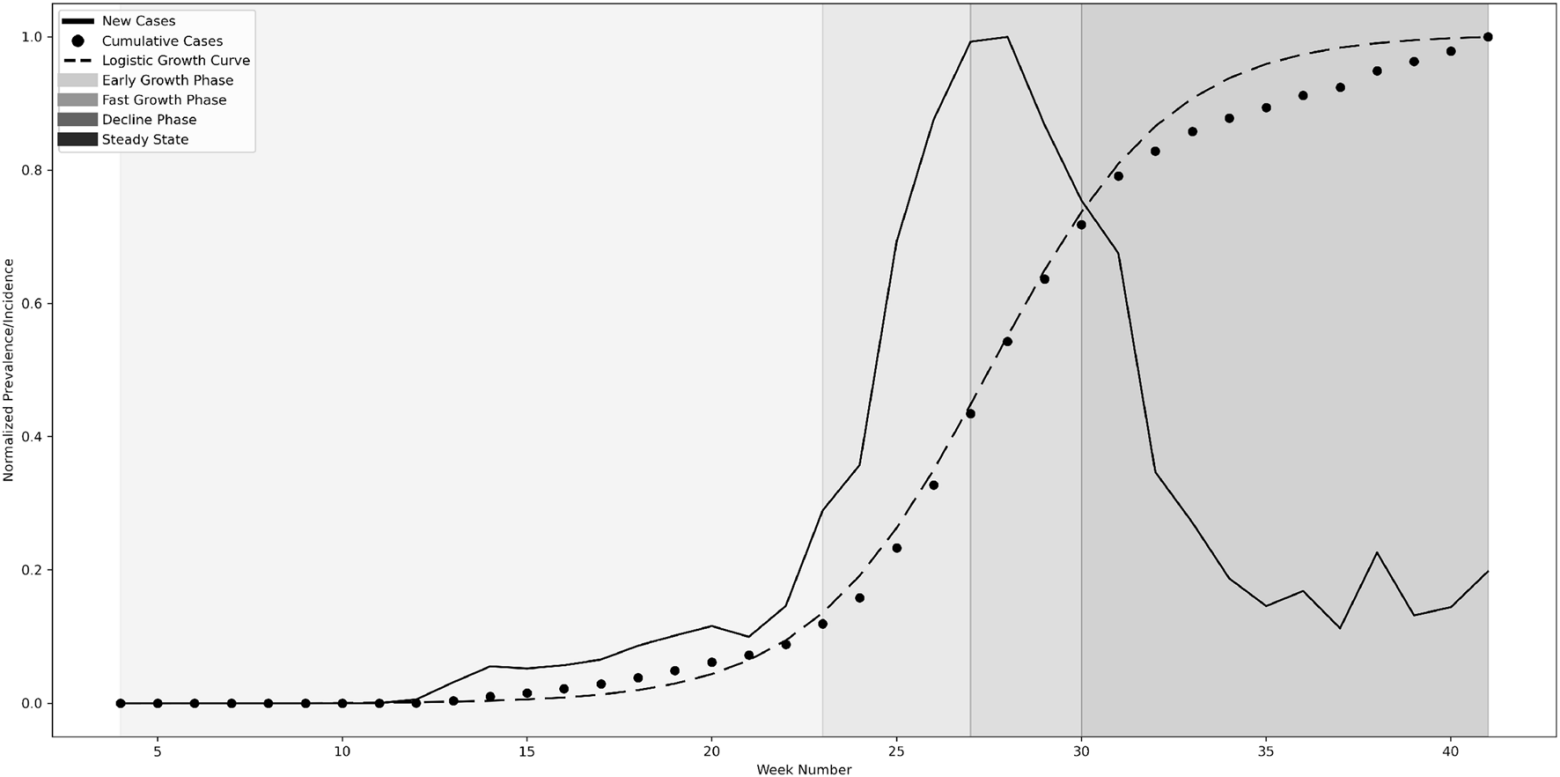
Logistic growth model applied to the state of Arizona.

We discretized the incidence growth rate of the pandemic into five phases based on the standard intervals obtained from a logistic growth curve^43,44^. Given the states have multiple peaks, we use a multi-peak-based logistic growth model from Batista et al.^43^ to obtain discrete phases. Phase-I is called the * early-growth phase* (or ascending) where (b) Phase-II is the * fast-growth phase* which falls between the end of the lag phase (or slow growth phase) and the peak (c) Phase-III is the *decline phase* where the cases decrease from fast-growth to steady-state, (d) Phase-IV – *steady-state* and finally (e) Phase-V is the *ending phase*. We illustrated the first 4 phases for the state of Arizona in Fig. 2; the fifth phase is not visible in the image.

The incidence growth can be envisioned as transitions between various growth phases. Once the incidence curve goes into fast-growth phase, the public health officials intervene to flatten the curve using warnings/outreach for people to stay home or promote face mask converting. The study considers both the current and future incidence phases for association rule mining. The current phase is part of the antecedent, and the future phase is the consequent/target variable with a lag of 4 weeks. Based on a preliminary analysis, we found that the mobility, reopening mandates, and other factors are correlated with the number of cases with a lag of 4 weeks.

We collected 25 weeks of data, June 1, 2020, to November 15, 2020, across all 50 states. Since the future incidence phase is lagged by four weeks, we ended up with 21 weeks of transactional data. The dataset thus has 1050 transactions, with each transaction corresponding to 21 weeks for each of the 50 states. An example rule would be, $Mask Usage: state- wide\, \& \,Current\,Phase: early- growth \Rightarrow Future\,Phase:early- growth$. This rule implies that when a state-wide mask mandate is active and the state is in the early-growth phase, the state would remain in the early-growth phase. Mask usage, current phase, and future phase are the attributes. State-wide and early-growth are the corresponding values for mask mandate and current incidence phase, respectively. The antecedents in the dataset are mask mandates, state re-openings, mobility levels, case levels, population density, and current incidence rate. The consequent or the target variable is the future incidence rate. In this analysis, we set the minimum support threshold to 0.01. This means that the combination of factors in the antecedent and the consequent should appear in at least ten transactions (ten weeks of data) to be considered important. This threshold could mean that the antecedent can appear across 10 weeks in a single state or 1 week across 10 states or any combination in between. The minimum confidence is 0.7, and the minimum lift is 1.

## Results

429 out of 55,125 relationships generated from the original transactions met the minimum threshold levels described in the Data and Methods section (support of 0.01, confidence of 0.7, and a lift value greater than 1). Each of these rules appeared in at least 10 transactions, i.e., 10 weeks of observations across the United States. With a confidence score of 0.7, each of the consequent (RHS) appears in at least 70% of the transactions with the antecedent (or the LHS). Finally, a high lift score (greater than 1) tells us that the factors in the antecedent are sufficiently positively correlated for deriving conclusions from the data.

**Table 2:** Top 5 association rules for different combinations of current and future phases, their support, confidence, lift, and chi-squared measures.

Association Rule	Support	Confidence	Lift	Chi-squared
**Continued early-growth**				
Mask Usage: state-wide & Current Phase: early-growth $\Rightarrow$ Future Phase: early-growth	0.11	0.97	3.16	273.15
Mask Usage: state-wide & Social Distancing: Phase 3 & Current Phase: early-growth $\Rightarrow$ Future Phase: early-growth	0.05	0.93	3.03	105.64
Mask Usage: None & Mobility Levels: Very High & Current Phase: early-growth $\Rightarrow$ Future Phase: early-growth	0.05	0.76	2.17	54.56
Mask Usage: state-wide & Case Level: Medium & Current Phase: early-growth $\Rightarrow$ Future Phase: early-growth	0.04	1.00	2.85	80.94
Mask Usage: state-wide & Mobility Level: High & Current Phase: early-growth $\Rightarrow$ Future Phase: early-growth	0.04	0.98	3.19	87.76
**Early-growth to fast-growth**				
Mask Usage: None & Case Level: Very Low & Current Phase: early-growth $\Rightarrow$ Future Phase: fast-growth	0.03	0.71	1.76	15.3
Mask Usage: None & Case Level: Very Low & Social Distancing: Phase 0 & Current Phase: early-growth $\Rightarrow$ Future Phase: fast-growth	0.02	0.75	1.85	12.15
Mask Usage: None & Case Level: Very Low & Social Distancing: Phase 0 & Population Density: Low & Current Phase: early-growth $\Rightarrow$ Future Phase: fast-growth	0.02	0.73	1.80	9.7
Mask Usage: None & Mobility Level: Very Low & Current Phase: early-growth $\Rightarrow$ Future Phase: fast-growth	0.01	1.00	2.47	17.85
Mask Usage: None & Mobility Level: Very Low & Social Distancing: Phase 0 & Current Phase: early-growth $\Rightarrow$ Future Phase: fast-growth	0.01	1.00	2.47	17.85
**Continued fast-growth**				
Mask Usage: state-wide & Current Phase: fast-growth $\Rightarrow$ Future Phase: fast-growth	0.16	0.77	1.89	145.86
Mask Usage: state-wide & Population Density: Low & Current Phase: fast-growth $\Rightarrow$ Future Phase: fast-growth	0.12	0.79	1.96	116.89
Mask Usage: None & Current Phase: fast-growth $\Rightarrow$ Future Phase: fast-growth	0.10	0.72	1.79	67.48
Mask Usage: None & Population Density: Low & Current Phase: fast-growth $\Rightarrow$ Future Phase: fast-growth	0.09	0.72	1.87	66.07
Mask Usage: state-wide & Social Distancing: Phase 1 & Current Phase: fast-growth $\Rightarrow$ Future Phase: fast-growth	0.05	0.71	1.76	34.04
**Continued Decline**				
Mask Usage: Countywide & Current State: Decline $\Rightarrow$ Future State: Decline	0.03	0.78	3.8	97.99
Mask Usage: Countywide & Population Density: Low & Current State: Decline $\Rightarrow$ Future State: Decline	0.03	0.94	4.09	97.72
Mask Usage: Countywide & Population Density: Low & Social Distancing: Phase 3 & Current State: Decline $\Rightarrow$ Future State: Decline	0.02	1.00	4.86	78.72
Mask Usage: Countywide & Social Distancing: Phase 3 & Current State: Decline $\Rightarrow$ Future State: Decline	0.02	0.83	4.05	51.21
Mask Usage: Countywide & Case Level: High & Current State: Decline $\Rightarrow$ Future State: Decline	0.02	0.82	3.98	51.59
**Steady-state to early-growth**				
Mask Usage: None & Current Phase: steady-state $\Rightarrow$ Future Phase: early-growth	0.03	0.90	2.94	51.39
Mask Usage: None Population Density: Low & Current Phase: steady-state $\Rightarrow$ Future Phase: early-growth	0.03	0.90	2.94	51.39
Mask Usage: None & Mobility Level: Very High & Social Distancing: Phase 0 & Current Phase: steady-state $\Rightarrow$ Future Phase: early-growth	0.02	0.94	3.09	35.2
Mask Usage: None & Social Distancing: Phase 0 & Current Phase: steady-state $\Rightarrow$ Future Phase: early-growth	0.02	0.94	3.09	35.2
Mask Usage: None & Mobility Level: Very High & Current Phase: steady-state $\Rightarrow$ Future Phase: early-growth	0.02	0.93	3.05	28.24

Table 2 shows the top 5 association rules for various combinations of current and future incidence phases. These rules show various factors that contributed to the infection growth pattern, which is represented as one of four phases (i.e., early-growth, fast-growth, decline, and steady-state). Of the 8 possible combinations between the current and the future incidence phases, we observe strong association rules that satisfy the minimum thresholds described above for 5 combinations: continued early-growth, early-growth to fast-growth, continued fast-growth, continued decline, and steady-state to early-growth. In Table 2, the first five rules highlight the circumstances where the incidence of cases stays constant, continuing in the same phase. The next five rules highlight scenarios where the incidence rate increases in the early-growth phase and transitions into the fast-growth phase. We also present the support, confidence, and lift values for each of these rules. These represent the rule’s coverage, strength, and predictive power, respectively, along with the chi-squared value of that rule. Given an antecedent and a consequent of a rule, the critical value of $\chi ^2$ is 3.841 for a significance of p<0.05^45^. A chi-squared value greater than 3.841 implies that the association between the antecedent and consequent in a rule is significant. All the association rules presented in Table 2 are significant.

We observed five combinations of current and future phases in the extracted association rules. The following are a summary of interesting observations:

1. *Continued Early-Growth * These rules represent the scenarios in which the number of cases continues to grow at a constant rate. The most important rule (i.e., 11% support and 97% confidence) shows that a state can remain in an early-growth phase even when there is a mask mandate. Another rule with lower support (5% support and 76% confidence) represents a scenario where states remain in the early-growth phase without a mask mandate and high mobility. In addition, the rules in the continued early-growth phase also demonstrate that states with a mask mandate, along with high mobility, medium-case levels, and phase-3 social distancing, will also continue in the early-growth phase.
2. * Early-Growth to Fast-Growth * Here, the number of cases increase rapidly, leading to an explosion in the number of new cases. The top 5 rules that contributed to the fast-growth phase from the early-growth phase have no mask mandates as the underlying common factor. Moreover, these rules have strong support and high confidence when no-mask is combined with low mobility, strict social distancing guidelines (i.e., phase 0), and a low number of cases.
3. *Continued Fast-Growth * When a state is in a fast-growth phase, we did not observe a specific combination of factors that lead to a decrease in the number of cases.
4. *Continued Decline * When case counts were decreasing, the top 5 rules have either a county-level or a state-level mask mandate. We observed this pattern alongside multiple factors (high mobility, high case levels, and relaxed social distancing guidelines).
5. * Steady-State to Early-Growth * When the states transitioned from a steady-state to the early-growth stage (indicating a resurgence in COVID 19 cases), we observed all the top 5 rules had a no-mask mandate. Other antecedents for these rules include a combination of a lower number of cases, strict social distancing guidelines, and very high mobility.

**Figure 3:**
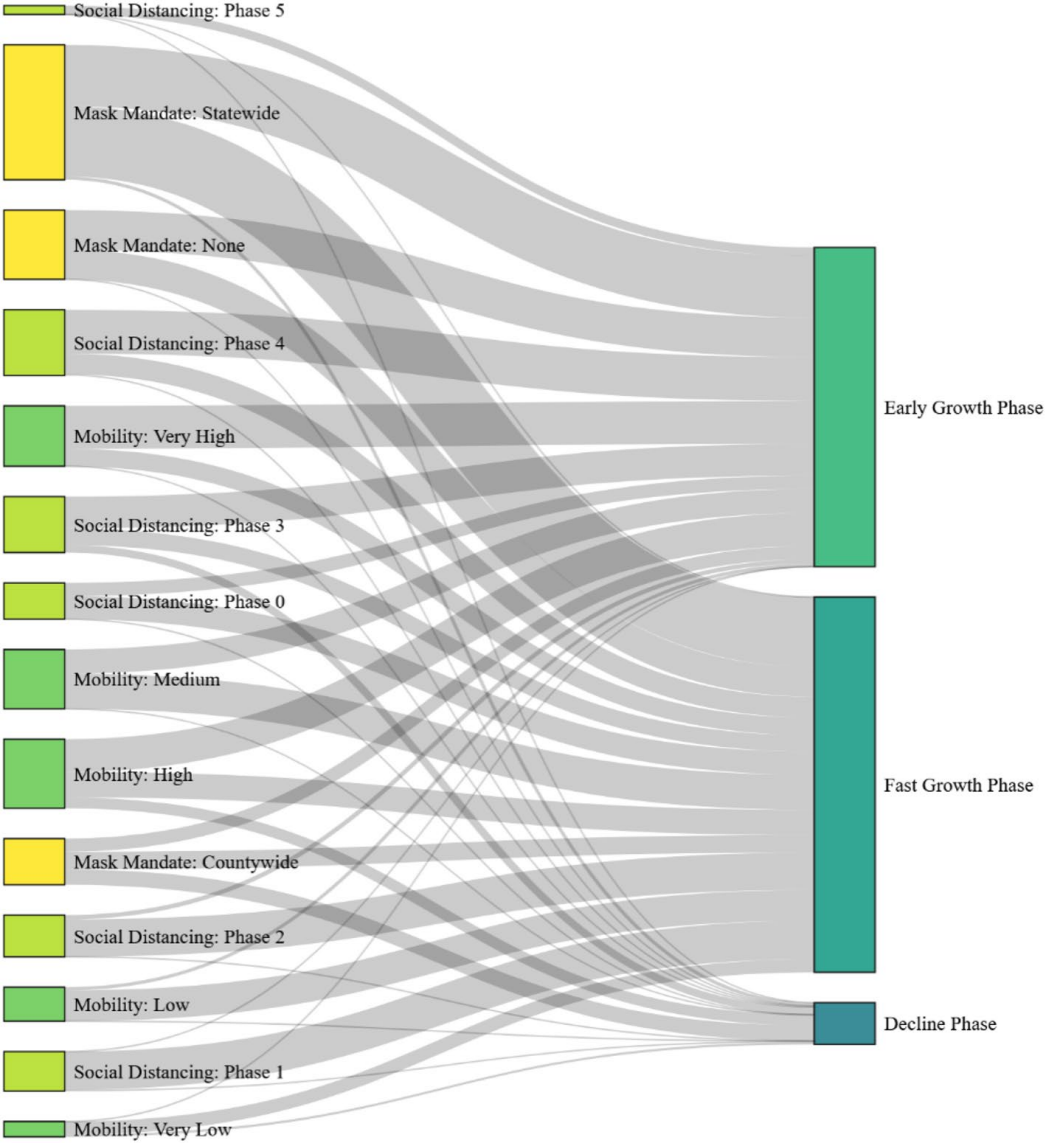
Association of various variables to the antecedent (future incidence curve of the pandemic).

We used a Sankey diagram to illustrate the combination of factors that contribute to different infection growth phases in Fig. 3. We present the contributing factors on the left and the resulting phase from the combination of contributing factors on the right. The width of the edge between the antecedent and the consequent represents the rules frequency for the given antecedent and consequent set. The flow lines show the relative strength of different factors (mask mandates, local mobility, population density, and social distancing orders) that contribute to the future incidence phase. The higher the number of rules for a particular variable, the larger the impact of that variable in affecting the outcome in the incidence. For example, in the case of state-wide face mask mandate, the highest number of rules (77 rules) are associated with the early-growth phase, followed by the fast-growth phase (66 rules), and the declining phase has the least number of rules (12 rules) in the dataset. The following are some interesting observations from Table 2.

- Rules with no mask mandate were only associated with either an early-growth phase (54.34%) or a fast-growth phase (45.65%). There were no rules with a no-mask mandate where the future incidence phase is a decline phase or a steady-state phase.
- In comparison, the rules with mask mandates (state-wide and countywide) were associated with all three future incidence phases: early-growth, fast-growth, and decline phases with 52.12%, 35.1%, and 12.76% rules in each phase, respectively.
- Reopening guidelines issued by the states were strongly associated with specific phases of the pandemic. Strict guidelines instituted during Phase 0 were always associated with rules in the early-growth and the fast-growth phases, as most states imposed strict lock-downs as the number of cases started to increase. On the other hand, the incidence of cases increased when these restrictions were relaxed. Phase 3 and 4 reopening guidelines led to a resurgence in the incidence (early-growth and fast-growth) in 87.74% of the rules, and a decrease in incidence was observed in 12.24% of the rules.
- Mobility has a considerable impact in determining the future phase of the pandemic. Lower mobility was associated with the early-growth phase, 3.2% of the total rules associated with low or very low mobility compared with 80.6% of rules leading to a fast-growth phase, and 16.12% of rules where the future phase is a decline phase. On the other hand, the rules with medium or higher mobility were associated mainly with future phases leading to early-growth, fast-growth, and decline phases 65.7%, 30.09%, and 3.3%, respectively. These distributions imply that lower mobility was associated with a decline in the number of cases, while higher mobility was associated with an increase in the number of cases.

## Discussion

COVID-19 policies with respect to vaccinations, mobility restrictions, shutdowns, mask mandates, etc., are currently the nation’s highest priorities towards saving lives and protecting the economy. Identifying and profiling the combination of policies that worked and did not work is important. This provides the necessary data for a rational decision support framework on how best to manage policies at the state level, given their diverse attributes. While the existing studies provide individual correlations, associations, forecasting, etc., they do not provide insights into effective combinations. The goal of our proposed method is to improve this understanding to aid policymakers in making the right decisions to help minimize spread while balancing convenience and economic growth priorities.

### Relationship between case-growth and mask mandates

**Figure 4:**
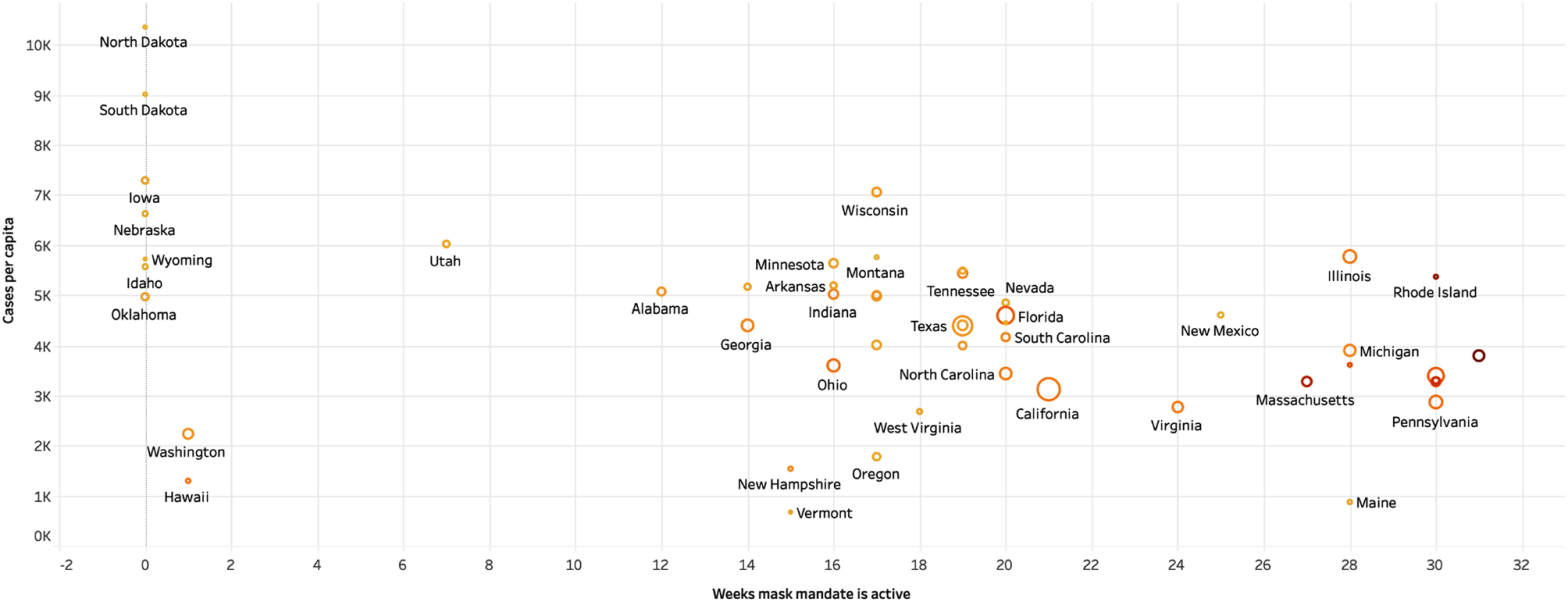
Relationship between the number of cases per capita and the number of weeks a mask mandate is active in a state.

Based on the association rules in Table 2, no mask mandates were always associated with an increase in the number of cases, and mask mandates were associated with a decrease in the number of cases. While it is not clear which specific measures led to a decrease in the number of cases, the mask mandates were always associated with a continued decline in the number of new cases. Most of the states issued a mask mandate when the number of cases was increasing rapidly, alongside stay-at-home orders. This observation is in line with earlier research showing that strong social distancing measures reduced the number of cases. However, the effect of mask mandates separate from social distancing measures is not apparent in the fast-growth phase. This was because the two measures were typically instituted together when the cases were increasing. For this reason, we cannot assess the differential contributions of these measures. We observed that the mask mandates were effective in the early-growth and decline phases of the pandemic. We also observed that the states that did not institute a mask mandate continued to see an increase in the number of cases for a longer duration than the states that did. Figure 4 shows the relationship between the number of cases per capita and the length of time the mask mandates were active in the different states. The color of the map shows the population density of a state, and the size shows the number of cases in that state. We observe that the longer the duration for which the mask mandates were active, the lower were the number of cases per capita. We also observed that states with high population densities that instituted a mask mandate had a lower number of cases per capita.

### Relationship between mobility and case-growth

Our results shown in both Table 2 and Fig. 3 indicate that mobility also impacts the incidence rate of the pandemic. The association rules indicate that increased mobility and a lack of mask mandates were associated with a resurgence of cases. A majority of the states in the United States successfully controlled the spread of the pandemic in spring and summer with strict social distancing guidelines and the resultant reduction in mobility. However, all the states had an increase in the number of cases in October and November, despite having issued mask mandates at state and county levels. This was likely related to increased mobility during this time period. In states that did not institute mask mandates, there was an increase in the number of cases irrespective of the mobility levels or the social distancing guidelines issued by the state and local authorities. By this, we surmise that social distancing and masking regulations were by themselves inadequate to reduce the number of new cases.

**Figure 5:**
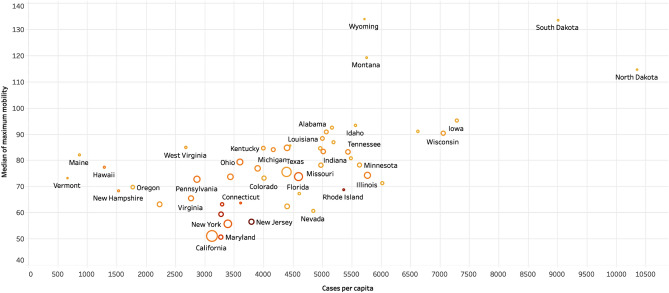
Impact of number of weeks mask mandate was active and the mobility on the number of cases per capita.

Figure 5 shows the relationship between the number of cases per capita and the median of maximum mobility for that state at a weekly level of granularity. The size of each marker shows the total number of cases, and the color indicates the number of weeks that state had a mask mandate. The states with mobility lower than 80 percent of the baseline had a lower number of cases per capita compared to states that had higher mobility. The states with the highest mobility, i.e., South Dakota, North Dakota, Wyoming, and Montana, were also the states with a considerably higher number of cases. These observations indicate that while mask mandates are essential, reducing the mobility of individuals and strict regulations on the businesses open also had a significant association with a reduction in the number of cases.

The states that did not institute mask mandates did not also impose strict social distancing guidelines or relaxed the guidelines earlier than most of the other states. These include states like South Dakota, Mississippi, North Dakota, and Utah. Both North Dakota and Utah imposed strict state-wide mask mandates in mid-November when the number of cases increased exponentially. Our results in Table 2 and Fig. 3 show the effect that various mask mandates, socials distancing guidelines, and mobility had on the change in the growth rate of the pandemic.

## Limitations and future work

We emphasize the limited scope of our analysis, as it is important to interpret these results with a clear understanding of the limitations with respect to both the data quality and the methodology.

Our data includes the start and end dates of various interventions by state and local authorities, but this does not help us measure the actual compliance to these measures. In the case of mask mandates issued at a county level, in a majority of the states, the population under the coverage of the mandates or recommendations is not known. We also did not consider several other conditions that affect growth in cases. For example, the analysis does not consider events such as holidays, weather conditions, congregation events, etc. Our assumptions about the incidence growth phase are based on the best fit from the logistic growth model.

In ARM, the choice of parameters (i.e., support and confidence thresholds) affect the rules generated^46^. If the thresholds are set too high, then we obtain very few rules. If the thresholds are set too low, we obtain too many rules. To make the analysis less susceptible to thresholds, we used the top 5 rules to study the impact of various factors to account for changes in phases of the pandemic. The discretization of variables also affects the type of rules generated. For instance, using just three classes (low, medium, and high) rather than five classes (very low, low, medium, high, and very high) produces a very different set of rules. We use five-class categorization using symmetric quantiles to discretize the variables and found them to yield better quality rules. In the future, a supervised discretization technique based on the strength of association rules can be used to further improve the quality of the rules generated. Future work can explore sensitivity analysis towards this goal. This approach provides a new direction to develop AI-based techniques that can provide policy recommendations for policymakers on various actions that could potentially decrease the number of new cases.

## Conclusion

We introduced a novel approach to analyze the effects of different non-pharmaceutical interventions to contain and manage the infection growth rate. The approach uses the association rule mining technique and discretization of infection growth phases, using a multi-peak logistic growth model. We made several interesting observations. For instance, there is a strong similarity between states that had strict mask mandates and reduced infection growth rates. Also, no difference was observed in terms of infection growth rate between state-wide versus county-wide mask mandates. Various other factors such as population density and mobility levels impacted the increase in the number of cases, highlighting the importance of local factors on the number of COVID-19 cases. These findings are important as the United States is trying to reach herd immunity through vaccination, while balancing against a growing resistance towards measures from various state level administrations and an exhausted population.

## Data Availability

The analysis code for this paper is available on GitHub at https://github.com/raviteja-bhupatiraju/AssociationMining_COVID19.
